# A ciência na construção da ignorância sobre a toxicidade de substâncias e ambientes nocivos

**DOI:** 10.1590/S0104-59702023000100019

**Published:** 2023-05-15

**Authors:** Bruno Capilé

**Affiliations:** i Pesquisador, Museu de Astronomia e Ciências Afins. Rio de Janeiro – RJ - Brasil brcapile@gmail.com

Há sessenta anos Rachel Carson (1962) denunciou, em *Primavera silenciosa* , o revés do desenvolvimento tecnológico da modernidade industrial na agricultura: a poluição química. A abordagem bélica de combate às pragas que se aproveitavam dos agroecossistemas desequilibrados das monoculturas resultou no uso indiscriminado do DDT, de organoclorados e outros agrotóxicos. A obra e a atuação de Rachel Carson incentivaram intenso ativismo ambiental nas décadas seguintes e levaram à proibição de algumas dessas substâncias no Norte global, bem como revelaram a irresponsabilidade científica do governo estadunidense junto à indústria química ( Lear, 1993 ).

Décadas depois, após outras denúncias da sutil violência da invisibilidade da contaminação química nos corpos humanos e nos ambientes, uma nova obra sobre o tema foi organizada pelos historiadores das ciências Ximo Guillem-Llobat e Agustí Nieto-Galan: a coletânea *Tóxicos invisibles: la construcción de la ignorancia ambiental* . A obra explora o modo como a história de algumas substâncias e ambientes tóxicos foi ocultada, de maneira deliberada ou passiva, muitas vezes com a conivência de cientistas de instituições públicas e corporações privadas. O esforço argumentativo e retórico desses textos deu visibilidade aos efeitos nocivos dessas substâncias e desses ambientes, assim como deu voz às vítimas da toxicidade. A atuação técnico-científica do Estado e das grandes empresas na ocultação dos riscos, ou, de forma mais assertiva, na elaboração de sofisticados mecanismos de construção de ignorância, dificultou a regulação adequada de produtos e espaços tóxicos. De muito interesse para o campo da história das ciências, a obra evidencia as tramas e os conflitos em que as comunidades científicas se inserem, ou são inseridas, contribuindo para desmitificar a suposta neutralidade científica.

Na introdução, o conceito de agnotologia (Proctor, Schiebinger, 2008) torna-se o fio condutor do livro para abordar a invisibilização da toxicidade. De acordo com os autores, o termo contempla o estudo da ignorância em três tipos taxonômicos. O primeiro seria a ignorância tradicional, o desconhecimento sobre determinado assunto. O segundo tipo é resultante de decisões seletivas da comunidade científica, em que alguns integrantes propõem resoluções que passivamente aceitam procedimentos e parâmetros de toxicidade com risco para a saúde humana e ambiental. Por fim, há a ignorância construída de maneira ativa e premeditada. Essa modalidade é feita diretamente pela comunidade científica em seus relatos para a mídia ou em relatórios técnicos, assim como por membros do governo ou das empresas com interesse em delinear estratégias para gerar informações duvidosas, criar incertezas ou manipular dados. A análise historiográfica desses dois últimos tipos de ignorância navega por uma tênue linha de interpretação de intencionalidades, entre a negligência involuntária e a deliberada.

Nove dos dez capítulos se referem à Espanha (e um ao Chile) e apresentam um país onde as substâncias químicas causaram e ainda causam sofrimento, com uma violência lenta sobre as pessoas, cujo processos de resistência são invisibilizados e assimétricos em relação a grupos hegemônicos, como grandes corporações ou Estados autoritários (assim como os governos de Franco ou Pinochet). Em todos os textos houve um claro posicionamento crítico para analisar e revelar os mecanismos sutis de ocultação dessas toxicidades pela prática e pelo discurso técnico-científico das empresas e instituições estatais.

A maioria dos capítulos é de integrantes do grupo de pesquisa Toxic Spain e derivou dos debates ocorridos principalmente na Escola de Primavera de História da Ciência, em Menorca, 2015. Tais preparo e organização trouxeram grande coesão e unicidade ao livro, articulando estilo de escrita, uso de conceitos e análise de fontes historiográficas. Os autores apostaram em caminhos interdisciplinares para contemplar tanto dados quantitativos e estatísticos resultantes de estudos de determinadas substâncias ou espaços enfermos quanto o estudo de testemunhos pessoais, jornais e documentários. Outro aspecto de coesão, além da narrativa da construção da ignorância e desses caminhos interdisciplinares e suas fontes, foi o uso da mídia visual no início de cada capítulo, com fotografias de jornais, charges, documentários e outros, apontando como o tema da toxicidade perpassava a sociedade espanhola, explicitando-o ou invisibilizando-o.

Dos dez capítulos, quatro concentram a análise em substâncias perigosas com alto potencial de toxicidade. O primeiro capítulo, “El vinu español y el espiritu alemán”, retrata como se deu a chegada do álcool industrial alemão em disputa com o vinho espanhol num complexo conflito discursivo entre elites quanto à sua toxicidade, em jornais, revistas e relatórios. Em “La (in)visibilización del riesgo de fumigaciones cianhídricas”, o autor explana como os técnicos exaltavam a eficácia do ácido cianídrico contra os insetos herbívoros nas oliveiras enquanto ocultavam o risco à vida dos trabalhadores rurais. Ainda no âmbito rural de combate às pragas, em “El escarabajo del marqués” o autor aponta como o uso do arsênico contra o besouro da batata antecedeu em décadas o amplo uso do DDT na época da ditadura de Francisco Franco (1939-1975). A invisibilização da toxicidade tornou-se projeto de governo e buscou legitimação junto aos técnicos e cientistas vinculados às instituições públicas, especialmente na proposta de limites de toxicidade para a vida humana. O capítulo “El ministro en bicicleta” expõe a participação do ministro franquista Laureano Lopez Rodó na flexibilização dos limites e padrões do risco nocivo dessas e outras substâncias no Congresso das Nações Unidas para o Meio Ambiente, em Estocolmo, em 1972.

Os demais seis capítulos contam histórias das zonas de sacrifício, espaços insalubres com alta toxicidade oculta em que uma lenta e invisível violência atinge vítimas igualmente invisíveis, cujo sofrimento não cabe nas estatísticas. Alguns desses dados tornaram-se secretos como assunto de Estado, por exemplo os dados médicos das vítimas de radioatividade em “Bañadores, detectores e ignorancia nuclear”. A ignorância seletiva por parte do poder público dava destaque a documentos que amenizavam os riscos enquanto omitia estudos científicos sobre a toxicidade, como a história da transformação do antigo lixão de Barcelona em um parque natural, em “La restauración del paisaje”. Esse mesmo paradoxo de criação de área protegida num espaço contaminado por resíduos tóxicos também é examinado no capítulo “Toxicidad e invisibilidade en la Albufera”, que analisa o modo como produtores de arroz se valeram de uma “fachada” verde para seus produtos, ocultando a intoxicação do ambiente e de seus consumidores. A estética e o uso das artes visuais marca a coletânea como um todo, mas em “Capitalismo químico y representaciones artísticas” a criação de obras de arte é colocada em evidência. Nesse texto, são mencionadas algumas das obras que valorizavam a estética da paisagem da histórica mina a céu aberto de Corta Atalaya, ressaltando a beleza, invisibilizando a toxicidade e legitimando a atividade mineradora, ao mesmo tempo que outras obras denunciavam de maneira mais proativa a contaminação química. As retóricas de oferta de emprego e desenvolvimento econômico eclipsaram os riscos da contaminação por cloro dos trabalhadores da fábrica Ercros, em “Desempleo o miseria”, e por cobre no Chile, em “Coreografias del abandono”.

Numa complexa e coesa história social da ciência, a obra contempla tanto a produção do conhecimento científico no âmbito das instituições e laboratórios como a divulgação desse conhecimento. E revela nessas histórias uma sociedade do risco ( Beck, 1992 ), em que a modernidade industrial cria produtos para consumidores, assim como substâncias e ambientes de risco para trabalhadores e cidadãos. A divulgação dos riscos, feita diretamente por parte da comunidade científica, resultou, em parte, em um novo contrato social: o de que vivemos em um mundo tóxico, e o risco de aí viver é aceito com a criação de normas e padrões para os limites de toxicidade.

## References

[B1] BECK Ulric (1992). Risk society: towards a new modernity.

[B2] CARSON Rachel (1962). Silent spring.

[B3] GUILLEM-LLOBAT Ximo, NIETO-GALAN Agustí (2020). Tóxicos invisibles: la construcción de la ignorancia ambiental.

[B4] LEAR Linda J (1993). Rachel Carson’s Silent Spring. Environmental History Review.

[B5] PROCTOR Robert N., SCHIEBINGER Londa (2008). Agnotology: the making and unmaking of ignorance.

